# Endovascular treatment and cognitive outcome after anterior circulation ischemic stroke

**DOI:** 10.1038/s41598-020-75609-1

**Published:** 2020-10-28

**Authors:** Simona Lattanzi, Michela Coccia, Alessandra Pulcini, Claudia Cagnetti, Federica Lucia Galli, Laura Villani, Serena Campa, Mauro Dobran, Gabriele Polonara, Maria Gabriella Ceravolo, Mauro Silvestrini

**Affiliations:** 1 Neurological Clinic, Department of Experimental and Clinical Medicine, Marche Polytechnic University, Via Conca 71, 60020 Ancona, Italy; 2 Neurorehabilitation Clinic, Department of Experimental and Clinical Medicine, Marche Polytechnic University, Ancona, Italy; 3 Clinic of Neuroradiology, Marche Polytechnic University, Ancona, Italy; 4 Clinic of Neurosurgery, Marche Polytechnic University, Ancona, Italy

**Keywords:** Medical research, Neurology

## Abstract

The impact of reperfusion therapies on cognition has been poorly explored and little knowledge exists. We explored the influence of endovascular treatment (EVT) on cognitive outcome in patients with anterior circulation ischemic stroke. Patients presenting with ischemic stroke due to anterior large vessel occlusion who underwent intravenous thrombolysis (IVT) alone or EVT plus IVT were recruited. Cognitive abilities were evaluated at 6 months from stroke through a neuropsychological test battery. A total of 88 patients with a mean age of 66.3 ± 12.9 years were included, of which 38 treated with IVT alone and 50 with IVT plus EVT. Compared to patients treated with IVT alone, patients who received EVT plus IVT performed significantly better at the neuropsychological tests exploring executive functions, attention, abstract reasoning, visuospatial ability, visual and verbal and memory. At multivariable regression analysis, the EVT was independently associated with the 6-month cognitive performance after the adjustment for age, sex, admission National Institutes of Health Stroke Scale score, systolic blood pressure, glucose level, Alberta Stroke Program Early CT score, side of stroke, site of occlusion, and Back Depression Inventory score [Stroop Test Word Reading: _adj_β = 13.99, 95% confidence interval (CI) 8.47–19.50, p < 0.001; Stroop Test Colour Naming: _adj_β = 6.63, 95% CI 2.46–10.81, p = 0.002; Trail Making Test-A: _adj_β = − 92.98, 95% CI − 153.76 to − 32.20, p = 0.003; Trail Making Test-B: _adj_β = − 181.12, 95% CI − 266.09 to − 96.15; p < 0.001; Digit Span Test Forward: _adj_β = 1.44, 95% CI 0.77–2.10, p < 0.001; Digit Span Test Backward: _adj_β = 1.10, 95% CI 0.42–1.77, p = 0.002; Coloured Progressive Matrices: _adj_β = 5.82, 95% CI 2.71–8.93, p < 0.001; Rey Complex Figure Test-Copy: _adj_β = 6.02, 95% CI 2.74–9.30, p < 0.001; Rey Complex Figure Test-Immediate recall: _adj_β = 6.00, 95% CI 2.34–9.66, p = 0.002; Rey Complex Figure Test-Delayed recall: _adj_β = 5.73, 95% CI 1.95–9.51, p = 0.003; Rey Auditory Verbal Learning Test-Immediate recall: _adj_β = 12.60, 95% CI 6.69–18.52, p < 0.001; Rey Auditory Verbal Learning Test-Delayed recall: _adj_β = 1.85, 95% CI 0.24–3.45, p = 0.025]. Patients treated with EVT plus IVT had better cognitive performance than patients treated with IVT alone at 6 months from anterior circulation ischemic stroke.

## Introduction

Cognitive impairment is a common consequence after stroke^1,2^. It is closely related to disability, dependency and institutionalization, and it is a major determinant of poor quality of life in stroke survivors^3–5^. So far, the impact of reperfusion therapies on cognition has been poorly explored and little knowledge exists. Indeed, physical recovery represents the main endpoint in stroke trials, whereas cognitive outcome is generally overlooked^6,7^.

The aim of this study was to investigate the effect of the endovascular treatment (EVT) on cognitive functioning in patients with ischemic stroke due to proximal arterial occlusion of the anterior circulation by comparing the 6-month neuropsychological performance in patients treated with intravenous thrombolysis (IVT) alone and IVT plus EVT.

## Results

**Figure 1:**
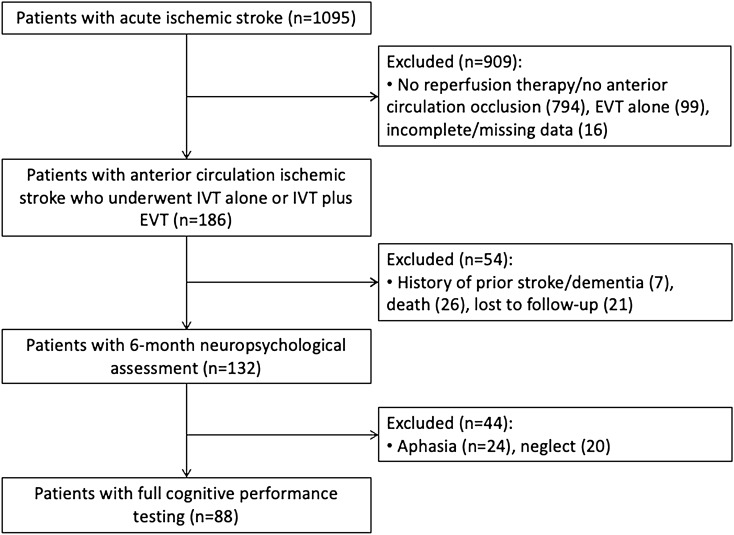
Patient selection flow diagram. *EVT* endovascular treatment, *IVT* intravenous thrombolysis.

A total of 186 out of 1095 patients admitted to our Stroke Unit for ischemic stroke underwent IVT alone or IVT plus EVT for a proximal arterial occlusion of the anterior circulation (Fig. 1). Fifty-four patients were excluded due to history of prior stroke/dementia (n = 7), death (n = 26), and unavailability of neuropsychological assessment as lost to follow-up (n = 21). The comparison of baseline characteristics between the patients with 6-month neuropsychological assessment and those who were excluded due to lack of follow-up did not show significant differences (Supplementary Table S1). Among the patients (n = 132) who underwent neuropsychological evaluation at 6 months from stroke, 44 patients were further excluded from the full cognitive performance testing since they presented with aphasia (n = 24) and neglect (n = 20). The characteristics of the patients excluded due to the impairment in language and visuo-spatial inattention domains are shown in Supplementary Table S2.

Accordingly, 88 patients were included in the analysis, of which 38 treated with IVT alone and 50 with IVT plus EVT. Patients did not receive EVT due to stroke occurrence before full implementation of EVT delivery at the site (n = 21), mild neurologic deficit at onset (n = 8), successful opening of occlusion/marked improvement of neurological deficit by IVT (n = 8), and very elderly (n = 1).

The mean age of the patients was 66.3 ± 12.9 years and 31 (35.2%) were females; 45 (51.1%) patients had right and 43 (48.9%) left hemisphere stroke. Baseline demographic and clinical characteristics of the study cohort according to the treatment group are shown in Table 1. No statistically significant differences in the prevalence of vascular risk factors and stroke severity emerged among the two groups, with the exception of serum glucose levels and NIHSS score at admission, which were higher among the patients treated with IVT plus EVT.

**Table 1:** Baseline characteristics of patients.

	IVT (n = 38)	IVT plus EVT (n = 50)	p value
**Demographics**			
Age (years)	67.2 (11.4)	65.6 (14.1)	0.562^a^
Male sex	29 (76.3)	28 (56.0)	0.048^b^
Education (years)	8 [5–13]	8 [8–13]	0.104^c^
**Clinical history**			
Current smoking	8 (21.1)	11 (22.0)	0.915^b^
Hypertension	25 (65.8)	31 (62.0)	0.714^b^
Diabetes mellitus	3 (13.2)	5 (10.0)	0.644^b^
Dyslipidaemia	23 (60.5)	22 (44.1)	0.124^b^
Coronary artery disease	4 (10.5)	10 (20.0)	0.229^b^
**Admission assessment**			
Systolic BP (mmHg)	150 [140–160]	150 [140–160]	0.352^c^
Serum glucose (mg/dl)	106 [89–120]	129 [109–140]	< 0.001^c^
NIHSS score	10.2 (5.7)	15.8 (4.0)	< 0.001^a^
ASPECTS value	9 [8–10]	8 [7–10]	0.158^c^
Location of intracranial occlusion			0.122^b^
Internal carotid artery	3 (7.9)	3 (6.0)	
^d^Internal carotid artery terminus	–	4 (8.0)	
Middle cerebral artery			
*First segment*	22 (57.9)	34 (68.0)	
*Second segment*	11 (29.0)	9 (18.0)	
Anterior cerebral artery A1	2 (5.3)	–	

Data are mean (SD) or median [IQR] for continuous variables, and n (%) for categorical variables.

*ASPECT* Alberta Stroke Program Early CT, *BP* blood pressure, *EVT* endovascular treatment, *IQR* interquartile range, *IVT* intravenous thrombolysis, *NIHSS* National Institutes of Health Stroke Scale, *SD* standard deviation.

^a^Two-sample t test.

^b^Chi-squared test.

^c^ Mann–Whitney test.

^d^Associated internal carotid artery and middle cerebral artery occlusion (tandem occlusion).

The scores obtained by the patients in the neuropsychological tests performed at 6 months from stroke are summarized in Table 2. Patients treated with IVT alone obtained lower (worse) scores at the SCWT, DST, CPM, RCFT-C, RCFT-I, RCFT-D, RAVLT-I and RAVLT-D and higher (worse) scores at the TMT-A and TMT-B. Patients in the IVT group had also higher (worse) scores on the BDI in comparison to patients treated with IVT plus EVT [8.0 (2.0–11.0) versus 3.5 (0.0–9.0); p = 0.096] and higher (worse) scores on the mRS [3.0 (1.0–4.0) versus 1.0 (0.0–2.0); p < 0.001] in comparison to patients treated with IVT plus EVT.

**Table 2:** Cognitive performance at 6 months from stroke.

	IVT (n = 38)	IVT plus EVT (n = 50)	p value
**Stroop test**			
Word reading	33.4 (16.0)	41.5 (11.1)	0.006^a^
Colour naming	18.4 (10.9)	23.2 (7.8)	0.018^a^
**Trail making test**			
Part A	90.5 [36.0–307.0]	36.0 [27.0–87.0]	0.005^b^
Part B	233.5 [57.0–562.0]	73.0 [41–227]	0.003^b^
**Digit span test**			
Forward	4.8 [3.1–5.3]	5.2 [4.3–5.8]	0.018^b^
Backward	3.2 [2.0–4.3]	4.1 [3.3–5.0]	0.005^b^
Coloured Progressive Matrices	25.0 [18.5–31.0]	28.8 [24.5–33.5]	0.016^b^
**Rey complex figure test**			
Copy	28.0 [21.4–30.6]	30.5 [27.0–31.5]	0.007^b^
Immediate recall	18.0 [12.6–24.6]	24.3 [18.4–28.1]	0.016^b^
Delayed recall	16.9 [8.7–20.6]	18.6 [13.7–26.5]	0.022^b^
**Rey auditory verbal learning test**			0.081^a^
Immediate recall	22.9 (11.3)	32.9 (13.7)	< 0.001^a^
Delayed recall	7.0 (4.1)	8.6 (3.0)	0.038^a^

Data are mean (SD) or median [IQR]. Higher values indicate worse performance for the Trail Making Tests and better performance for all the other cognitive tests.

*EVT* endovascular treatment, *IVT* intravenous thrombolysis.

^a^Two-sample t test.

^b^Mann–Whitney test.

The results of the regression analysis are shown in Table 3. The acute stroke treatment resulted a significant predictor of 6-month cognitive outcome being the EVT plus IVT associated with better cognitive performances, before and after the adjustment for potential confounding factors (Table 3). None of the multivariate models suffered from collinearity (variance inflation factors ranged from 1.10 to 2.05).

**Table 3:** Associations between 6-month cognitive performance and stroke treatment.

Dependent variable	Unadjusted			Adjusted^a^		
	β	95% CI	p	β	95% CI	p
Stroop test word reading	8.16	2.40 to 13.91	0.006	13.99	8.47 to 19.50	< 0.001
Stroop test colour naming	4.81	0.85 to 8.77	0.018	6.63	2.46 to 10.81	0.002
Trail making test-A	− 113.49	− 167.85 to − 59.14	< 0.001	− 92.98	− 153.76 to − 32.20	0.003
Trail making test-B	− 174.63	− 254.64 to − 94.62	< 0.001	− 181.12	− 266.09 to − 96.15	< 0.001
Digit span test forward	0.79	0.19 to 1.39	0.011	1.44	0.77 to 2.10	< 0.001
Digit span test backward	0.97	0.37 to 1.58	0.002	1.10	0.42 to 1.77	0.002
Coloured progressive matrices	3.81	0.75 to 6.87	0.015	5.82	2.71 to 8.93	< 0.001
Rey complex figure test-copy	3.52	0.50 to 6.53	0.023	6.02	2.74 to 9.30	< 0.001
Rey complex figure test-immediate recall	4.38	0.75 to 8.01	0.019	6.00	2.34 to 9.66	0.002
Rey complex figure test-delayed recall	4.44	1.18 to 7.69	0.008	5.73	1.95 to 9.51	0.003
Rey auditory verbal learning test-immediate recall	10.06	4.61 to 15.51	< 0.001	12.60	6.69 to 18.52	< 0.001
Rey auditory verbal learning test-delayed recall	1.60	0.09 to 3.11	0.038	1.85	0.24 to 3.45	0.025

Values are from linear regression models with cognitive scores as dependent variables.

*ASPECT* Alberta Stroke Program Early CT, *BDI* Back Depression Inventory, *BP* blood pressure, *CI* confidence interval, *NIHSS* National Institutes of Health.

^a^Adjustment for age, sex, admission NIHSS score, systolic BP, glucose level, ASPECT score, side of stroke, site of occlusion, BDI score.

## Discussion

The main finding of this study was the better 6-month cognitive outcome observed in patients with stroke due to proximal large vessel occlusion who underwent IVT combined with EVT than IVT alone. At the follow-up visit, patients treated with IVT plus EVT performed better in the tests exploring executive functions and attention, abstract reasoning, constructive ability, and visuospatial and verbal memory.

The early recanalization of the occluded vessels is the main mechanism underlying the beneficial effects of the reperfusion therapies: it can restore flow to the ischemic penumbra and prevent its transformation into necrotic tissue^8^. Significant differences, however, exist between pharmacological intravenous fibrinolysis and mechanical endovascular clot removal as the responsiveness of large thrombi to enzymatic digestion is quite poor, whereas EVT can rapidly remove proximal clots^9–12^. The early recanalization through EVT has demonstrated to be more efficacious in lowering the risk of mortality, reduces the severity of disability and increases the rate of functional independence in comparison to standard medical care in patients with strokes due to large vessels occlusions^13^. The current study extends the findings of the recent randomized controlled trials and provides evidence that the EVT has the potentiality to favorably influence the post-stroke cognitive recovery. Indeed, the mRS—the most commonly used instrument to assess clinical outcome in stroke trials—relies mostly on physical functions and under-represents cognitive abilities. Detailed neuropsychological assessments can be beneficial both in stroke trials and clinical practice^14–16^. First, a broad spectrum of cognitive changes occurs after stroke and multiple domains and complex neuropsychological abilities are typically compromised. Second, cognitive deficits are prevalent also in patients with the most favorable clinical recovery and no apparent functional disability. Third, even milder cognitive deficits can have impact on independent functioning, occupational abilities and quality of life^17,18^. Additionally, items that are often affected by stroke, as processing speed, calculation, and praxis, are not included in current screening measures, and require a comprehensive investigation to be fully explored^19^.

In the prespecified secondary analysis of the REVASCAT (Endovascular Revascularization With Solitaire Device Versus Best Medical Therapy in Anterior Circulation Stroke Within 8 h) trial, patients randomized to thrombectomy plus best medical treatment rather than best medical treatment alone performed better in the TMT-A and TMT-B at 3 months and 1 year after stroke due to anterior circulation proximal arterial occlusion^20^. It is however worth to notice that cognitive outcome was evaluated with one single test, which focused on executive functioning, and the lack of a comprehensive neuropsychological battery did not allow drawing conclusions on other cognitive domains. In addition, as motor dexterity is required to perform the TMT, the results and their interpretation could be biased by co-occurring impairment in motor function of the upper arm; the high proportion of participants who did not complete the task in the requested time and, hence, received the maximum time score, may have masked performance variability among the most severely impaired patients and allowed to identify significant differences between treatment groups only in functionally independent patients. Finally, symptoms of depression were collected indirectly and not included in multivariable analysis.

Recently, Xu et al. found that patients with mild to moderate anterior circulation infarct who received mechanical thrombectomy at broadened therapeutic window had higher scores in Mini-Mental State Examination and Montreal Cognitive Assessment tests at the 90 days follow-up than those receiving standard therapy treatment^21^. Although both tests can assess multiple cognitive abilities, they represent global screening tools for detecting cognitive impairment rather than instruments to thoroughly evaluate the neuropsychological domains. Moreover, the very small differences in total scores observed between the treatment arms and the lack of data about the individual items of both tests make difficult the clinical interpretation of the findings.

The main strengths of the current study included the comparison of patients who underwent treatment with rt-PA alone versus rt-PA combined with mechanical thrombectomy, which allowed to minimize the heterogeneity in baseline patients’ characteristics and time onset-to-treatment and, hence, estimate the actual effect deriving from the EVT. The exclusion of patients presenting with aphasia or neglect from the comprehensive neuropsychological assessment allowed to obtain a more reliably evaluation of the neurocognitive performance as results in cognitive tests are significantly affected and confounded by the presence of deficits in the domains of language and visuo-spatial inattention^22,23^. Finally, the 6-month interval from stroke to follow-up can be considered a sufficiently long time for the acute stroke effects to subside^24,25^, and the real-world setting of the research increased the generalizability of the findings to routine clinical practice. Nonetheless, some study shortcomings need to be considered. The retrospective analysis of data collected at a single academic center could have led to selection bias and findings need to be validated in independent cohorts. The relatively small sample size prevented sub-group analyses and no data on health-related quality of life have been considered at the follow-up. Additionally, no specific information regarding treatment success, including reperfusion rates, follow-up infarct volumes or hemorrhage rates have been considered, and further studies designed to comprehensively assess these parameters as well as the relationship between infarct location and test scores are warranted.

## Conclusion

The growing number of stroke survivors has increased the interest in long-term sequelae and prediction of cognitive outcome. In this regard, treatment with EVT plus IVT can result in better cognitive performance than IVT alone in patients with anterior circulation ischemic stroke.

## Methods

### Study participants

We retrospectively identified consecutive patients with anterior circulation ischemic stroke, admitted to the Stroke Unit of the Marche Polytechnic University (Ancona, Italy) from January 2012 to June 2019, who were treated with IVT alone and IVT plus EVT, and underwent neuropsychological assessment at 6 months from the index event as part of routine care. The site serves in the region as referral comprehensive stroke center (hub) for mechanical thrombectomy for large vessel occlusion according to a drip-and-ship organizational model of stroke care. Patients were included if they had intracranial proximal arterial occlusion in the anterior circulation [intracranial carotid artery (ICA) or middle cerebral artery (M1/M2) or anterior cerebral artery (A1/A2)] demonstrated by vascular imaging (computed tomographic angiography or magnetic resonance angiography or digital subtraction angiography), received IVT within 4.5 h and started EVT within 6.0 h after the onset of stroke. IVT consisted of the administration of recombinant tissue plasminogen activator (rt-PA) at the dose of 0.9 mg/kg (maximum 90 mg; 10% bolus followed by a 60-min infusion). EVT consisted of mechanical thrombectomy with aspiration catheters alone, stent-retrievers alone, or both, depending on occlusion type and interventionist’s choice.

Data about demographic, vascular risk factors, medical history, baseline stroke severity according to the National Institutes of Health Stroke Scale (NIHSS) score^26^, admission systolic blood pressure (BP) and serum glucose were collected, as previously detailed^27–29^. The ischemic lesion extension was estimated according to the Alberta Stroke Program Early CT Score (ASPECTS) on head computed tomography (CT) performed in emergency prior to IVT administration^30^. Patients with a neurological or psychiatric history, pre-stroke modified Rankin Scale (mRS)^31^ score > 2, patients who did not perform the neuropsychological evaluation at 6 months from stroke and those who presented aphasia or neglect at the 6-month evaluation according to the Aphasia Neuropsychological Exam (ANE) (language)^32^ and Apples Cancellation Test (ACT) (visuo-spatial inattention)^33^ were not included.

### Neuropsychological assessment

The neuropsychological assessment was administered by a trained examiner at a single session 6 months after stroke using standardized cognitive tests at the Clinic of Neurorehabilitation of the Marche Polytechnic University as part of clinical care. Scores obtained in the following neuropsychological tests were considered in the current analysis as representative of different cognitive domains: Stroop Colour and Word Test (SCWT)^34^, Trail Making Test parts A (TMT-A) and B (TMT-B)^35^, Digit Span Test (DST) (executive functions and attention)^36^, Coloured Progressive Matrices (CPM) (abstract reasoning)^37^, Rey Complex Figure Test Copy (RCFT-C) (visuospatial ability), Rey Complex Figure Test immediate (RCFT-I) and delayed recall (RCFT-D) (visual memory)^38^, Rey Auditory Verbal Learning Test immediate (RAVLT-I) and delayed recall (RAVLT-D) (verbal memory)^39^. All test scores were corrected according to normative values; the score ranges of the cognitive tests are summarized in Supplementary Appendix-SI. Post-stroke depressive symptoms and functional abilities were assessed with the Beck Depression Inventory (BDI)^40^ and mRS^31^.

### Statistical analysis

Values were presented as mean ± standard deviation (SD) or median (interquartile range [IQR]) for continuous variables and as the number (%) of subjects for categorical variables. Univariate comparisons were made through the Student t test, Mann–Whitney test, or Chi-squared test, as appropriate. Linear regressions were performed to assess the influence of treatment (IVT alone versus IVT plus EVT) on scores obtained in each cognitive test, adjusting for pre-specified potential confounding factors (age, sex, admission NIHSS score, systolic BP, glucose level, ASPECT score, side of stroke, site of occlusion, BDI score). The collinearity between exposure variables was assessed with the variance inflation index. Results were considered significant for p values < 0.05 (two sided). Data analysis was performed using STATA/IC 13.1 statistical package (StataCorp LP, Texas, USA).

### Standard protocol approvals, registrations, and patient consents

The study was approved by the Ethics Committee of the Marche Polytechnic University and conducted according to the Declaration of Helsinki. Informed consent was obtained from any patient or the legal representative.

## Supplementary information

Supplementary Information

## Data Availability

Anonymized data will be shared by request from any qualified investigator.
