# Supplementary material for: Endovascular treatment and cognitive outcome after anterior circulation ischemic stroke
Source: Sci Rep. 2020 Oct 28;10:18524. doi: 10.1038/s41598-020-75609-1 (PMC7595128; doi:10.1038/s41598-020-75609-1)
Supplement: Supplementary file 1 — Supplementary Information [file 41598_2020_75609_MOESM1_ESM.pdf]

Scientific Report - Electronic Supplementary Material

Endovascular treatment and cognitive outcome after anterior circulation ischemic stroke

Simona Lattanzi[1], Michela Coccia[2], Alessandra Pulcini[1], Claudia Cagnetti[1], Federica Lucia Galli[2], Laura Villani[2], Serena Campa[3], Mauro Dobran[4], Gabriele Polonara[3], Maria Gabriella Ceravolo[2], Mauro Silvestrini[1]

[1]Neurological Clinic, Department of Experimental and Clinical Medicine, Marche Polytechnic University, Ancona, Italy

[2]Neurorehabilitation Clinic, Department of Experimental and Clinical Medicine, Marche Polytechnic University, Ancona, Italy.

[3]Clinic of Neuroradiology, Marche Polytechnic University, Ancona, Italy.

[4]Clinic of Neurosurgery, Marche Polytechnic University, Ancona, Italy.

Correspondence to: Simona Lattanzi, Neurological Clinic, Department of Experimental and Clinical Medicine, Marche Polytechnic University, Ancona, Italy; e-mail: alfierelattanzisimona@gmail.com

p3: Table e-1. Baseline characteristics of patients according to the availability of cognitive assessment

p4: Table e-2: Baseline characteristics of patients excluded due to aphasia and neglect

p5: e-Appendix-I

Table e-1. Baseline characteristics of patients according to the availability of cognitive assessment

	Cognitive assessment (n=132)	Lost to follow-up (n=21)	p value
**Demographics**			
Age (years)	66.8 (13.1)	68.2 (13.2)	0.637[a]
Male sex	81 (61.4)	14 (66.7)	0.642[b]
Education (years)	8 [5-13]	11 [8-13]	0.533[c]
**Clinical history**			
Current smoking	32 (24.2)	6 (28.6)	0.670[b]
Hypertension	82 (62.1)	15 (71.4)	0.411[b]
Diabetes mellitus	17 (12.9)	3 (14.3)	0.859[b]
Dyslipidaemia	60 (45.5)	10 (47.6)	0.853[b]
Coronary artery disease	19 (14.4)	3 (14.3)	0.990[b]
**Admission assessment**			
Systolic BP (mmHg)	150 [140-160]	150 [135-150]	0.282[c]
Serum glucose (mg/dl)	119 [102-142]	113 [91-135]	0.246[c]
NIHSS score	13.6 (5.4)	12.8 (5.8)	0.514[a]
ASPECTS value	9 [8-10]	9 [8-10]	0.390[c]
Location of intracranial occlusion			0.420[b]
Internal carotid artery	10 (4.8)	1 (7.6)	
*Internal carotid artery terminus	12 (9.1)	4 (19.1)	
Middle cerebral artery			
First segment	86 (65.2)	10 (47.6)	
Second segment	21 (15.9)	5 (23.8)	
Anterior cerebral artery A1	3 (2.3)	1 (4.8)	

Data are mean (SD) or median [IQR] for continuous variables, and n (%) for categorical variables.

*Associated internal carotid artery and middle cerebral artery occlusion (tandem occlusion).

[a]Two-sample t test. [b]Chi-squared test. [c] Mann-Whitney test.

Abbreviations: ASPECT= Alberta Stroke Program Early CT, BP=blood pressure, IQR=interquartile range, NIHSS=National Institutes of Health Stroke Scale, SD=standard deviation.

Table e-2. Baseline characteristics of patients excluded due to aphasia and neglect

	**Aphasia/Neglect (n=44)**
**Demographics**	
Age (years)	67.8 (13.4)
Male sex	24 (54.6)
Education (years)	8 [5-13]
**Clinical history**	
Current smoking	13 (29.6)
Hypertension	29 (65.9)
Diabetes mellitus	7 (15.9)
Dyslipidaemia	15 (34.1)
Coronary artery disease	5 (11.4)
**Admission assessment**	
Systolic BP (mmHg)	148 [140-160]
Serum glucose (mg/dl)	138 [100-157]
NIHSS score	14.0 (5.1)
ASPECTS value	9 [8-10]
Location of intracranial occlusion	
Internal carotid artery	4 (9.0)
*Internal carotid artery terminus	8 (18.2)
Middle cerebral artery	
First segment	30 (68.2)
Second segment	1 (2.3)
Anterior cerebral artery A1	1 (2.3)

Data are mean (SD) or median [IQR] for continuous variables, and n (%) for categorical variables.

*Associated internal carotid artery and middle cerebral artery occlusion (tandem occlusion).

Abbreviations: ASPECT= Alberta Stroke Program Early CT, BP=blood pressure, IQR=interquartile range, NIHSS=National Institutes of Health Stroke Scale, SD=standard deviation.

Appendix – I. Score ranges of cognitive tests

Cognitive Test	Range (raw score)	Adjusting variables	Range (adjusted score)	Pathological values
**Stroop Test**	Number of right answers in 30 seconds	Age, sex and education		
**Word reading**				≤28
**Colour Naming**				≤11
**Trail Making Test-A**	Time in seconds	Age, education		>93
**Trail Making Test-B**	Time in seconds	Age, education		>282
**Digit Span Test Forward**	2 to 9	Age, education	1.3 to 10.5	<4.3
**Digit Span Test Backward**	2 to 8	Age, education	1.2 to 9.6	<2.7
**Coloured Progressive Matrices**	0 to 36	Age, education	-4.0 to 43.5	≤17.5
**Rey Complex Figure Test**		Age, education		
- Copy	0 to 31		-1.4 to 32.8	≤26.9
- Immediate Recall	0 to 31		-3.8 to 35.9	≤16.8
- Delayed Recall	0 to 31		-3.9 to 36.3	≤16.0
**Rey Auditory Verbal Learning Test**		Age, education		
- Immediate Recall	0 to 75		-12.7 to 87.2	≤28.5
- Delayed Recall	0 to 15		-3.9 to 18.8	≤4.7
